# The Treatment of Syphilis, with Special Reference to the Best Methods of Administering Mercury*Abstract of an original paper by the author in *The Lancet* (London, Eng.), October 19, 1901.

**Published:** 1902-01

**Authors:** Winfield Ayres

**Affiliations:** Genito-Urinary Surgeon, Bellevue Hospital, O. D. P., New York; Instructor in Genito-Urinary Diseases in New York University and Bellevue Hospital Medical College; Instructor in Genito-Urinary Diseases in the New York Post-Graduate Hospital, Etc.


### The Treatment of Syphilis, With Special Reference to the Best Methods of Administering Mercury.*

BY WINFIELD AYRES, M. D.,
Genito-Urinary Surgeon, Bellevue Hospital, O. D. P., New York; Instructor in Genito-Urinary Diseases in New York University and Bellevue Hospital Medical College; Instructor in Genito-Urinary Diseases in the New York Post-Graduate Hospital, Etc.

The author calls to mind the facts that mercury has been used in the treatment of syphilis for over 400 years, and there are few physicians, today, who do not use it in some form. Although the method of treatment with mercury is still discussed, he is firmly of the opinion that there is no hope of eradicating the disease unless the full dose is given constantly for something like three years. The treatment should begin just as soon as the diagnosis can be made. There is no ground for supposing that enucleation of the chancre has the effect of aborting the disease. If a positive diagnosis cannot be made from the appearance of the initial lesion, general tonic treatment should be instituted.

In some cases the protiodide controls the symptoms, but in the majority it is of very little use. Experiments with Mercurol were conducted at Bellevue Hospital, for eight and a half months, with 180 cases; the histories of ninety-five of these are recorded. The remainder could not be kept under observation and are, therefore, passed over. The dosage of the Mercurol, regulated either by reaching the point of tolerance or control of the disease, varied from one-half to six grains. In sixty-four of the ninety-five cases the disease was controlled as follows: in two weeks, 8; three weeks, 12; four weeks, 14; five weeks, 6; six weeks, 5; seven weeks, 2; two months, 8; ten weeks, 2; three months, 5; and four months, 1. The remainder are marked thus: decidedly improved, 17; improved, 8; no improvement in two weeks, 3; no improvement in four weeks, 1; and no improvement in three months, 2. The latter were all dispensary patients and it is uncertain whether they took their medicine regularly.

The writer states that his plan was to increase the dose steadily from one grain until the symptoms were controlled, or until there was a slight tendency on the part of the teeth and gums to become tender. If the symptoms were not controlled before the physiological effect of the Mercurol made itself felt, small doses of potassium

*Abstract of an original paper by the author in *The Lancet* (London, Eng.), October 19, 1901.

iodide were added, and in every case where the Mercurol was taken according to directions, with the exceptions noted above, the symptoms were controlled.

In sixty-seven out of the ninety-five cases tabulated, no other medicine than Mercurol was given. In fifteen out of the remaining twenty-eight, the addition of iodide of potassium was found to be sufficient to control the disease, while in six others the addition of an iron tonic sufficed for this purpose.

The cases are not reported at length, but a few of the more remarkable results and some cases in which other medicines failed to control the disease are briefly mentioned.

Case 1 had been taking bichloride for one month with very little improvement. Under Mercurol, three grains maximum dosage, the symptoms were under control in five weeks.

Case 2 had been under biniodide of mercury (one-sixteenth of a grain) and potassium iodide (five grains), which caused iodism. His symptoms were controlled in one month under half a grain of Mercurol.

In case 3 unguentum hydrargyri had failed to control the disease. The patient was put on Mercurol and the dosage pushed up to six grains three times a day. The disease was thoroughly under control in seven weeks.

Case 4 had been on three-eighths of a grain of biniodide of mercury and twenty grains of potassium iodide for two months. The medicine caused nausea and vomiting. Having been put on Mercurol and the dosage gradually increased to five grains three times a day, the symptoms were controlled in three weeks.

Case 5 had been taking hydrargyrum bichloride (one-twelfth of a grain) three times a day, under which an eruption on his face had faded, but the eruption on his body still persisted. His symptoms disappeared in two weeks under a maximum dose of three grains of Mercurol three times a day.

Case 6 had been on bichloride of mercury (three-sixteenths of a grain) for three months, in spite of which he had palmar syphilide of an eczematous variety. All appearances of the disease disappeared after he had been one month on Mercurol, his maximum dose being three grains three times a day.

Case 7 had been taking one-quarter of a grain of Mercurol and fifteen grains of potassium iodide, with the result that the eruption had decidely improved, though not to the extent that it should have done. There were thickened red patches on the face, covered with scaly eruptions. The symptoms almost entirely disappeared within three weeks under a maximum dosage of five grains of Mercurol three times a day and fifteen grains of potassium iodide.

Case 8 had been treated with inunctions of mercury, under which the eruptions disappeared, but the pains in the bones still persisted. He was relieved in three weeks under a maximum dosage of four grains of Mercurol three times a day.

Case 9 had been taking other forms of mercury for six months. The form which had done him most good was bichloride. Yet one-fifth of a grain did not entirely control the disease. He had been taking that for two months when he was placed on Mercurol. The dosage in his case was pushed up to six grains three times a day, and at the end of seven weeks all his symptoms had disappeared.

Case 10 had been taking medicine off and on for two years, but his symptoms never disappeared entirely. After being two weeks on Mercurol (two grains three times a day) with the addition of potassium iodide, all symptoms had disappeared.

Ayres, in conclusion, states that he uses Mercurol in his private practice to the exclusion of all other drugs. His experience is that he gets better results. He has found no form in which mercury can be given with such good results as in that of Mercurol.

